# Functional Performance of Different Venous Limb Options in Simulated Neonatal/Pediatric Cardiopulmonary Bypass Circuits

**DOI:** 10.21470/1678-9741-2018-0074

**Published:** 2018

**Authors:** Luiz Fernando Caneo, Gregory S. Matte, Daniel Peres Guimarães, Guilherme Viotto, Marcelo Mazzeto, Idagene Cestari, Rodolfo A. Neirotti, Marcelo B. Jatene, Shigang Wang, Akif Ündar, João Chang Junior, Fabio B. Jatene

**Affiliations:** 1 Cardiovascular Surgery Division, Instituto do Coração, Hospital das Clínicas da Faculdade de Medicina da Universidade de São Paulo (InCor-HCFMUSP), São Paulo, SP, Brazil.; 2 Department of Cardiac Surgery, Boston Children's Hospital, Boston, MA, USA.; 3 Clinical Professor of Surgery and Pediatrics, Emeritus Michigan State University, MI, USA.; 4 Pediatric Cardiovascular Research Center, Department of Pediatrics; Public Health Sciences; Surgery and Bioengineering, Penn State Health Milton S. Hershey Medical Center, Penn State College of Medicine, Penn State Health Children's Hospital, Hershey, PA, USA.; 5 Department of Industrial Engineering, FEI University Center, São Paulo, Brazil.

**Keywords:** Cardiopulmonary Bypass, Pediatrics, Oxygenators, Membranes

## Abstract

**Objective:**

Hemodilution is a concern in cardiopulmonary bypass (CPB). Using a smaller dual tubing rather than a single larger inner diameter (ID) tubing in the venous limb to decrease prime volume has been a standard practice. The purpose of this study is to evaluate these tubing options.

**Methods:**

Four different CPB circuits primed with blood (hematocrit 30%) were investigated. Two setups were used with two circuits for each one. In Setup I, a neonatal oxygenator was connected to dual 3/16" ID venous limbs (Circuit A) or to a single 1/4" ID venous limb (Circuit B); and in Setup II, a pediatric oxygenator was connected to dual 1/4" ID venous limbs (Circuit C) or a single 3/8" ID venous limb (Circuit D). Trials were conducted at arterial flow rates of 500 ml/min up to 1500 ml/min (Setup I) and up to 3000 ml/min (Setup II), at 36°C and 28°C.

**Results:**

Circuit B exhibited a higher venous flow rate than Circuit A, and Circuit D exhibited a higher venous flow rate than Circuit C, at both temperatures. Flow resistance was significantly higher in Circuits A and C than in Circuits B (*P*<0.001) and D (*P*<0.001), respectively.

**Conclusion:**

A single 1/4" venous limb is better than dual 3/16" venous limbs at all flow rates, up to 1500 ml/min. Moreover, a single 3/8" venous limb is better than dual 1/4" venous limbs, up to 3000 ml/min. Our findings strongly suggest a revision of perfusion practice to include single venous limb circuits for CPB.

**Table:** 

Abbreviations, acronyms & symbols	
A-V	= Arterio-venous
ALF	= Arterial line filter
CPB	= Cardiopulmonary bypass
CVR	= Cardiotomy venous reservoir
GME	= Gaseous microemboli
GSD	= Gravity siphon drainage
ID	= Inner diameter
IVC	= Inferior vena cava
OR	= Operating room
USB	= Universal serial bus
VAVD	= Vacuum-assisted venous drainage

## INTRODUCTION

Cardiopulmonary bypass (CPB) is commonly utilized during surgical repair for congenital heart defects. The CPB circuit prime hemodilutes the patient once CPB is initiated. Limited hemodilution is known to provide the benefits of decreasing blood viscosity and improving microcirculatory flow^[^^1^^]^. However, hemodilution is also associated with a number of adverse side effects, including decreased plasma colloidal oncotic pressure, increased total body water, and coagulation abnormalities^[^^2^^,^^3^^]^. In consideration of these issues, perfusionists typically minimize the CPB circuit prime volume so as not to cause excessive hemodilution^[^^4^^-^^6^^]^. Other intraoperative techniques such as conventional ultrafiltration during CPB and modified ultrafiltration at the end of CPB are also important to minimize hemodilution and reduce the requirement for transfusions^[^^7^^]^. These are central concerns in pediatric cardiac surgeries since the bypass circuit prime volume tends to be larger than the patient's own circulating blood volume. In neonates, the CPB circuit prime may be as much as 200-300% of the patient's blood volume^[^^8^^]^.

The bypass circuit prime volume comprises the prime volume of primary components, including the oxygenator, cardiotomy venous reservoir (CVR), arterial pump head, arterio-venous (A-V) loop, arterial line filter (ALF), hemoconcentrator, and sampling lines^[^^7^^]^. The prime volume of most disposable components is constant when devising a CPB circuit. However, some aspects of the circuit are less standardized: the length, the inner diameter (ID), and at some centers, the number of venous lines utilized when bicaval cannulation is required. In addition to the number of venous lines used, the bypass circuit venous component can further vary with the drainage technique employed - gravity siphon drainage (GSD) *versus* vacuum-assisted venous drainage (VAVD)^[^^7^^]^. The use of VAVD is quite common as it can provide adequate venous drainage with smaller ID tubing, but it does not come without a downside risk. In fact, VAVD has been shown to increase the potential for gaseous microemboli (GME)^[^^9^^,^^10^^]^. While the effect of GME on overall pediatric patient outcomes is unclear^[^^11^^]^, most clinicians agree that, intuitively, we should minimize GME on bypass since the adult literature supports their negative impact on patient outcomes after cardiac surgery^[^^12^^,^^13^^]^. Therefore, while minimizing venous line tubing ID and maximizing the use of VAVD would decrease bypass circuit prime volume, other important considerations must be taken into account.

Finally, the selection of a venous line tubing has an important impact on venous drainage during bypass. Venous limb tubing is typically upsized compared with the patient's size owing to the increased kinetic potential of tubing sizes with larger internal diameters. Specific flow limitations for each size, dual or single limb venous circuits, are not well defined since table height relative to reservoir height, venous limb length, and reservoir construction vary across institutions^[^^7^^]^. Adequate venous drainage is essential for the optimal conduct of perfusion and this is, in large part, a function of the flow specifications for the venous limb. Inadequate venous drainage can result in edema and organ dysfunction^[^^14^^,^^15^^]^.

We currently employ three different circuits at the Heart Institute, University of São Paulo Medical School, Brazil. We categorize our circuits according to the sizes of the single arterial limb and the dual venous limbs, in this order. They are defined as neonatal (3/16" x 3/16" x 3/16"), pediatric (1/4" x 1/4" x 1/4") and adult (3/8" x 3/8" x 3/8"), since it is common practice in Brazil to provide individual venous drainage lines to each cava for bicaval cannulation. This has been a unique standard clinical practice for decades which deserved an evaluation.

The objective of this study was to evaluate venous limb options currently in use and to compare resistance and maximum flow rate capacity on the venous side of simulated CPB circuits in order to better qualify a current practice for anticipated bypass flow rates up to 3000 ml/min. A prime volume comparison of the different circuits was also done.

## METHODS

### Experimental Circuits

**Fig. 1:**
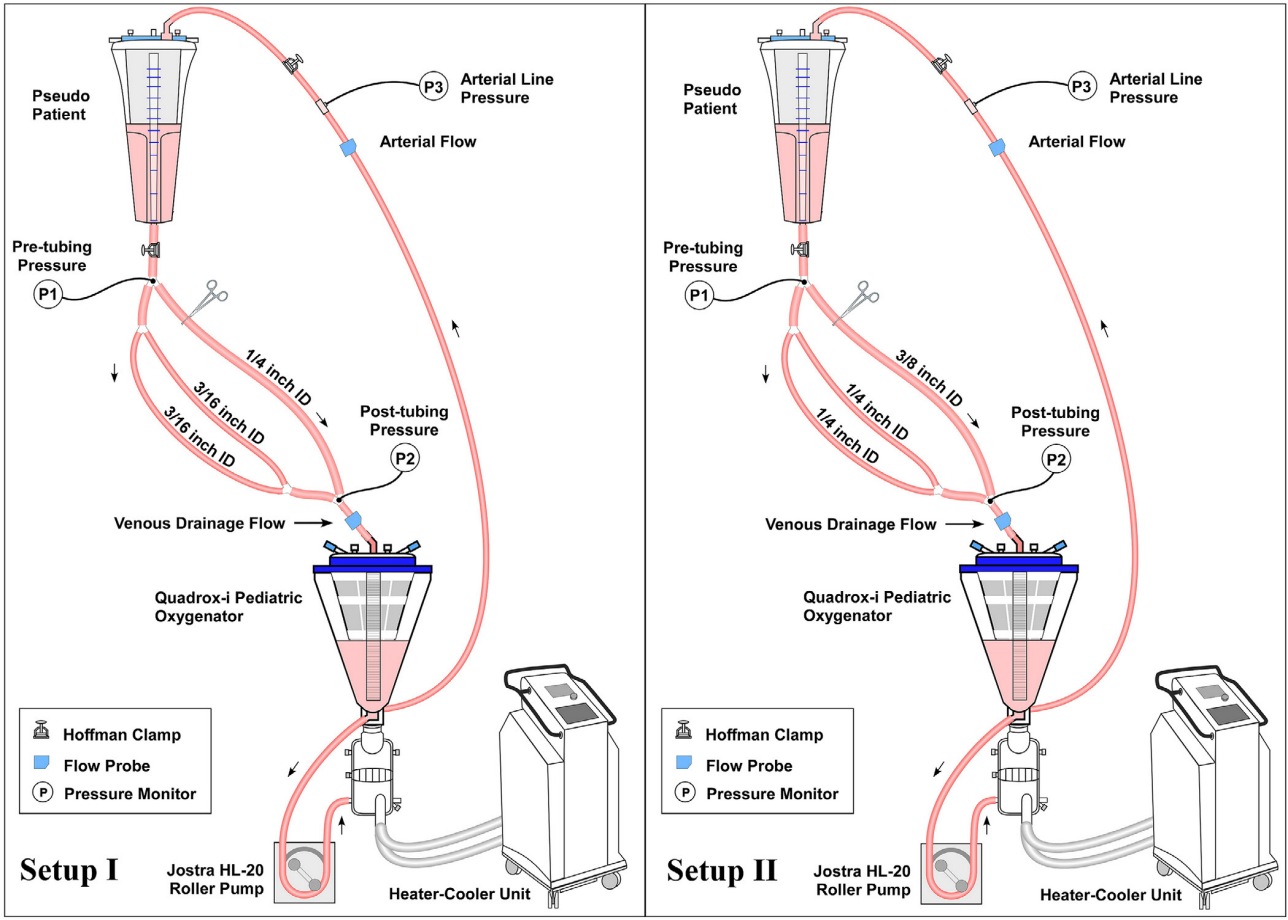
Setup I allows for testing Circuits A and B. Setup II allows for testing Circuits C and D. Heater-cooler units allowed for experiments to be done at 35°C and 28°C. Hoffman clamp on the circuit arterial limb allowed for a constant post-cannula pressure.

Circuit designs employed in this study simulated pediatric CPB and utilized the standard equipment in clinical use at the Heart Institute, University of São Paulo Medical School. The experimental circuit included Maquet (Maquet Cardiopulmonary AG, Rastatt, Germany) hardware, with a Jostra HL-20 roller pump and an HCU-20 heater-cooler system. The pseudopatient consisted of a 2000 ml capacity hardshell reservoir (Maquet Cardiopulmonary AG, Rastatt, Germany). The pseudopatient reservoir level was located 80 cm above the CVR and it was connected to options for venous tubing. Setup I included two 3/16" venous limbs and one 1/4" venous limb running from the pseudopatient to the CVR (Figure 1, Setup I). Setup II included two 1/4" venous limbs and one 3/8" venous limb running from the pseudopatient to the CVR (Figure 1, Setup II). Venous limb lengths were standardized to 120 cm. Maquet disposable oxygenator-reservoirs included either their Neonatal or Pediatric options. The arterial pump head for all test conditions included 150 cm of 1/4" ID tubing. A Hoffman clamp was placed at the distal end (just before the pseudopatient reservoir) of the arterial limb to maintain a constant post arterial cannula pressure during all trials. The CPB circuit was first primed with lactated Ringer's solution (Baxter, São Paulo, Brazil) and then packed red blood cells were added to achieve a circuit hematocrit of 30%. The venous reservoir level was kept at 200 mL for both oxygenators-reservoirs in use.

### Experimental Design

Table 1 lists the four circuits tested: A) two 3/16" ID venous limbs, B) a single 1/4" ID venous limb, C) two 1/4" ID venous limbs, and D) a single 3/8" ID venous limb. Circuits A, B and C included 1/4" arterial limbs whereas Circuit D included a 3/8" arterial limb.

**Table 1:** Venous limb circuit test specifications. Volume was measured using the circuit tubing tested in Setups I, II, and III.

Circuit tested	Venous Limb Size (ID)	Venous Limb Length (cm)	Total Venous Limb Prime Volume (ml)	Difference between Circuits
A	Two 3/16"	120	42	A-B = 5 ml
B	One 1/4"	120	37	
C	Two 1/4"	120	74	C-D = - 10 ml
D	One 3/8"	120	84	

To evaluate the performance of these circuits we used two different setups as shown in Figure 1 (Setups I and II).

Setup I was used to test circuits A and B at flow rates of 500 ml/min to 1500 ml/min in 500 ml/min increments, with Maquet Neonatal oxygenator-reservoir. We adjusted the Hoffman clamp for position A or B to test each venous option independently. Setup II was used for circuits C and D at flow rates between 1500 ml/min and 3000 ml/min in 500 ml/min increments, with Maquet Pediatric oxygenator-reservoir. We adjusted the Hoffman clamp for position C or D to test each venous option independently. The blood level of the pseudopatient was kept at 80 cm above the CVR in all experiments. Arterial line pressure (P3) was maintained at 50 mmHg during all trials. Experiments were conducted at 36°C and 28°C. Data were electronically collected.

**Fig. 2:**
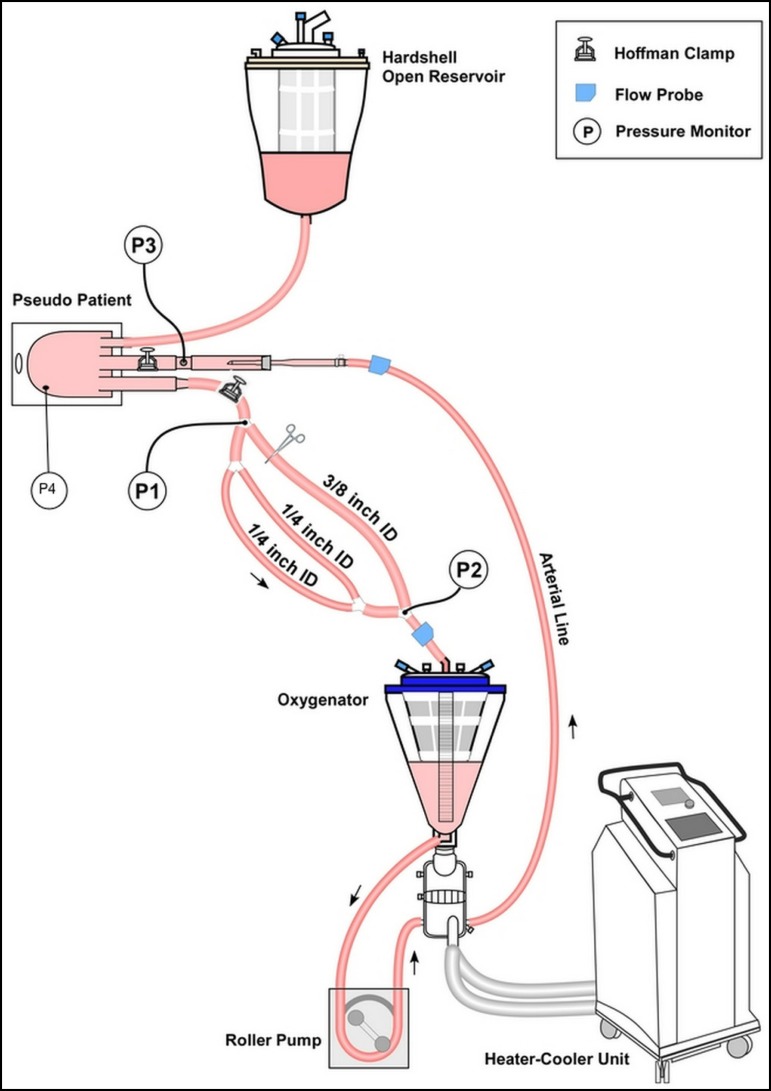
Setup III for testing Circuits C and D using a soft bag as pseudopatient. During this experiment, venous pressure was kept at a constant range of 3 to 4 mmHg, simulating a controlled venous pressure more similar to a clinical scenario. Heater-cooler units allowed for experiments to be done at 35°C and 28°C. Hoffman clamp on the circuit arterial limb allowed for a constant post-cannula pressure of 50 mmHg.

A second experiment was done using a 1600 ml capacity soft bag (Medtronic, Minneapolis, MN, USA) simulating the pseudopatient to test Circuits C and D in a different condition (Figure 2, Setup III). Setup III was used to test Circuits C and D with controlled venous pressure at flow rates between 1500 ml/min and 3000 ml/min in 500 ml/min increments, with Maquet Pediatric oxygenator-reservoir. A Hoffman clamp was placed near the distal end of the arterial line to maintain an arterial line pressure (P3) of 50 mmHg during all trials. The CPB circuit was primed with lactated Ringer's solution, and then packed red blood cells were added into the circuit to maintain the blood hematocrit at 30%. The reservoir venous pressure was kept at 3 to 4 mmHg, simulating the pseudopatient's venous pressure. The venous pressure was controlled using an open hardshell reservoir and a Hoffman clamp at the experimental venous limb. The total priming volume of the circuit was 2600 mL (Circuits C and D), including the pseudopatient's volume. We adjusted the Hoffman clamp for Setup D, then we repeated the experiment without any adjustments to Setup C. Experiments were conducted under normothermia (36°C) and hypothermia (28°C), separately. The entire process was repeated six times for each unique combination.

### Data Acquisition

Two Transonic ultrasound flow probes (Transonic Systems, Inc., Ithaca, NY, USA) were used for each set of test conditions. One flow probe was located at the venous inlet to the CVR and the other was located before arterial cannula, as shown in Figure 1. Three Edwards TruWave disposable pressure transducers (Edwards Lifesciences Corp., Irvine, CA, USA) were placed. The first transducer was located at the beginning of the venous limb (P1), the second was at the venous limb insertion to the CVR (P2), and the third was at the pre-arterial cannula site (P3). Pressure transducers were connected to pressure monitors CPB-100 (Bioengineering Division, InCor, São Paulo, Brazil). Pressure monitors and flowmeter outputs were connected to a DataQ DI-710 data acquisition device (DataQ, Akron, OH, USA) and then connected to a computer via universal serial bus (USB) port. WinDaq data acquisitions software (DataQ, Akron, OH, USA) was used to record real-time data at 1000 samples per second per channel. A 30 s segment of pressure and flow waveforms was recorded at all sites.

### Calculating Venous Line Resistance

Venous line resistance of each tubing set was calculated using the following equation (Tables 2, 3, and 4).

$$\mathrm{Venous}\;\mathrm{line}\;\mathrm{resis}\tan\mathrm{ce}\;\left(\mathrm{wood}\;\mathrm{units}\right)=\frac{\left(P2-P1\right)\left(\mathrm{mmHg}\right)}{\mathrm{Venous}\;\mathrm{flow}\;\left(L/\min\right)}$$

**Table 2:** Flow rate, pressure, and resistance of Setup I (Circuits A: two 3/16" and B: one 1/4").

Temperature (°C)	Group	Circuit	Venous flow (ml/min)	B-A (ml/min)	P1 (mmHg)	P2 (mmHg)	P2-P1 (mmHg)	Resistance (Wood unit)
36	500 ml/min	A	515.0±0.3	__	-15.5±0.0	-6.2±0.0	9.3±0.0	18.1
		B	538.9±0.6	23.9±0.9	-15.6±0.0	-8.7±0.0	6.9±0.0	12.7
	1000 ml/min	A	1013.9±1.0	__	-15.3±0.0	5.7±0.0	21.0±0.0	20.7
		B	1087.8±0.6	73.9±0.7	-15.1±0.0	1.2±0.0	16.2±0.0	14.9
	1500 ml/min	A	1516.5±1.4	__	-15.0±0.0	21.1±0.0	36.1±0.0	23.8
		B	1728.5±0.6	211.9±1.4	-14.7±0.0	15.9±0.0	30.6±0.0	17.7
28	500 ml/min	A	523.0±0.2	__	-15.7±0.0	-4.9±0.0	10.9±0.0	20.8
		B	548.1±0.3	25.1±0.3	-15.8±0.0	-7.6±0.0	8.2±0.0	14.9
	1000 ml/min	A	1014.0±0.7	__	-15.3±0.0	8.1±0.0	23.5±0.0	23.1
		B	1125.8±0.8	111.8±1.0	-15.1±0.0	3.3±0.0	18.4±0.0	16.3
	1500 ml/min	A	1518.2±0.8	__	-15.0±0.0	24.4±0.0	39.3±0.0	25.9
		B	1768.4±1.4	250.2±1.4	-14.6±0.0	19.0±0.0	33.6±0.0	19.0

**Table 3:** Flow rate, pressure, and resistance of Setup II (Circuits C: two 1/4" and D: one 3/8").

Temperature (°C)	Group	Circuit	Venous flow (ml/min)	D-C (ml/min)	P1 (mmHg)	P2 (mmHg)	P2-P1 (mmHg)	Resistance (Wood unit)
36	1500 ml/min	C	1525.7±1.5	__	-14.5±0.0	0.1±0.0	14.6±0.0	9.6
		D	1681.4±2.1	155.7±0.8	-14.3±0.0	-7.5±0.0	6.8±0.0	4.1
	2000 ml/min	C	2022.6±1.4	__	-13.3±0.1	8.8±0.0	22.1±0.0	10.9
		D	2328.0±0.4	305.4±1.2	-12.3±0.0	-2.0±0.0	10.3±0.0	4.4
	2500 ml/min	C	2516.0±0.6	__	-11.9±0.0	19.1±0.0	30.9±0.0	12.3
		D	3152.9±0.8	636.9±0.7	-9.8±0.0	6.0±0.0	15.8±0.0	5.0
	3000 ml/min	C	3054.7±0.6	__	-10.4±0.0	31.8±0.0	42.2±0.0	13.8
		D	4393.3±11.4	1338.6±11.6	-5.3±0.1	20.5±0.1	25.8±0.2	5.9
28	1500 ml/min	C	1528.4±2.0	__	-14.1±0.0	1.2±0.0	15.2±0.0	10.0
		D	1714.3±0.5	185.9±2.4	-13.8±0.0	-6.9±0.0	6.9±0.0	4.0
	2000 ml/min	C	2008.7±0.2	__	-13.0±0.0	9.7±0.0	22.8±0.0	11.3
		D	2354.2±0.9	345.5±0.8	-11.9±0.0	-1.2±0.0	10.7±0.0	4.5
	2500 ml/min	C	2506.2±0.6	__	-11.3±0.0	21.2±0.0	32.5±0.0	13.0
		D	3217.0±4.5	710.8±4.4	-8.6±0.0	8.2±0.0	16.8±0.0	5.2
	3000 ml/min	C	3021.4±2.8	__	-9.5±0.0	34.2±0.0	43.7±0.1	14.5
		D	4505.3±10.6	1483.9±12.6	-3.5±0.0	24.8±0.1	28.3±0.1	6.3

**Table 4:** Flow rate, pressure, and resistance of Setup III (test from Circuit D to C).

Temperature (°C)	Group	Circuit	Venous flow (ml/min)	D-C (ml/min)	P1 (mmHg)	P2 (mmHg)	P2-P1 (mmHg)	Resistance (Wood unit)
36	1500 ml/min	C	1418.3±0.4	__	-14.8±0.0	-1.6±0.0	13.2±0.0	9.3
		D	1537.4±1.0	119.0±1.0	-14.5±0.0	-8.3±0.0	6.2±0.0	4.0
	2000 ml/min	C	1804.5±0.6	__	-13.9±0.0	4.9±0.0	18.8±0.0	10.4
		D	2033.7±1.6	229.2±2.0	-13.2±0.1	-4.5±0.1	8.8±0.0	4.3
	2500 ml/min	C	2140.4±1.4	__	-13.0±0.0	11.4±0.0	24.4±0.0	11.4
		D	2508.6±0.2	368.3±1.4	-11.7±0.0	-0.1±0.0	11.6±0.0	4.6
	3000 ml/min	C	2451.2±1.2	__	-12.0±0.0	18.0±0.0	30.1±0.0	12.3
		D	3031.6±0.7	580.5±1.6	-10.1±0.0	5.0±0.0	15.1±0.0	5.0
28	1500 ml/min	C	1394.4±0.5	__	-14.5±0.0	-1.0±0.0	13.5±0.0	9.7
		D	1534.1±0.7	139.7±0.9	-14.4±0.0	-8.3±0.0	6.1±0.0	4.0
	2000 ml/min	C	1772.0±1.8	__	-13.7±0.0	5.5±0.0	19.2±0.0	10.8
		D	2015.9±0.4	243.9±2.1	-13.1±0.0	-4.2±0.0	8.8±0.0	4.4
	2500 ml/min	C	2116.2±1.4	__	-12.9±0.0	12.4±0.0	25.3±0.0	11.9
		D	2515.1±1.3	398.9±2.6	-11.3±0.0	0.7±0.0	12.0±0.0	4.8
	3000 ml/min	C	2397.9±0.8	__	-11.8±0.0	18.7±0.0	30.6±0.0	12.8
		D	3000.9±1.4	603.0±1.0	-9.6±0.0	5.8±0.0	15.4±0.0	5.1

### Statistical Analysis

A linear mixed-effects model was fit to continuous hemodynamic outcomes to compare tubing sizes (*e.g*., 1/4" and 3/16") and temperatures (*e.g*., 28°C and 36°C) within specific flow rates. The linear mixed-effects model is an extension of linear regression that accounts for the within-subject variability inherent in repeated measures designs. In this study, the repeated factor is the location in the simulated system. For each outcome, P values were adjusted for multiple comparisons testing using Tukey-Kramer procedure. All hypotheses tests were two-sided and all analyses were performed using SAS software, version 23 (SAS Institute, Inc., Cary, NC, USA).

## RESULTS

### Venous Limb Prime Volumes

The total volume necessary to fill 120 cm tubing of the venous limb was measured for each circuit option with results shown in Table 1.

### Venous Line Resistance and Flow Rate

The results for Circuits A and B using Setup I are shown in Table 2. Results for Circuits C and D using Setups II and III are shown, respectively, in Tables 3 and 4.

### Circuits A and B

Setup I compared dual 3/16" venous limbs (Circuit A) *versus* a single 1/4" venous limb (Circuit B) as shown in Table 2. The resistance across the circuit venous limb was assessed as well as the set pump flow rate *versus* the measured venous flow rate. Venous drainage was better with a single 1/4" venous line than with dual 3/16" venous lines, as indicated by a higher venous flow rate and a lower venous resistance at flow rates of 500 ml/min to 1500 ml/min, for both sets of temperature condition. Though, finding that the dual 3/16" circuit was less favorable at 1500 ml/min may be academic, as most clinicians would not limit inferior vena cava (IVC) flow to a single 3/16" venous line at such flow rate with a dual venous limb circuit. The IVC flow is typically thought to provide two-thirds of the return to the heart and this experimental design doesn't account for that. The 1/4" venous circuit had an advantage over the dual 3/16" venous limb, with small savings in prime volume (Circuit B has 5 ml less than Circuit A).

### Circuits C and D

Setup II compared dual 1/4" venous limbs (Circuit C) *versus* a single 3/8" venous limb (Circuit D) as shown in Table 3. The single 3/8" venous circuit had a higher flow at both temperature conditions with a slightly increased limitation at 28°C. The single 3/8" venous circuit had an apparent advantage over the dual 1/4" venous limb with a clinically insignificant 10 ml (Circuit C has 10 ml of prime volume less than Circuit D) prime volume increase.

The results of using Setup III to test Circuits C and D with controlled venous pressure and flow up to 3000 ml are shown in Table 4. A higher achievable flow rate was also evident, although less marked, with a single 3/8" tubing in the venous limb compared with the dual 1/4" venous limb.

### Venous Line Resistance

**Fig. 3:**
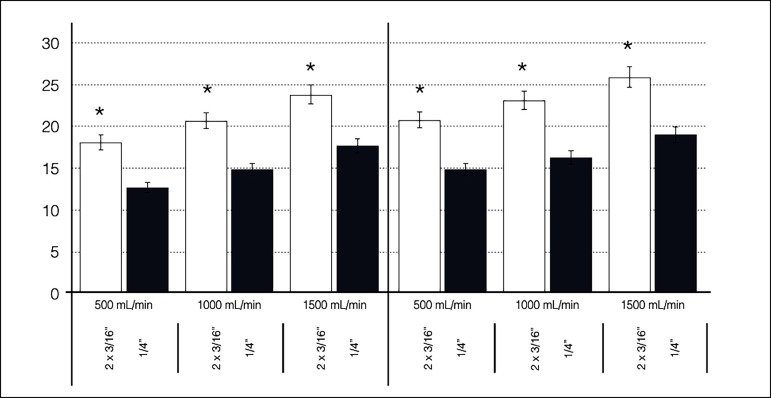
Venous line resistance according to the arterial flow rate observed in Circuits A and B, in both normothermia and hypothermia (setup I), showing significant difference between them (*P<0.001).

**Fig. 4:**
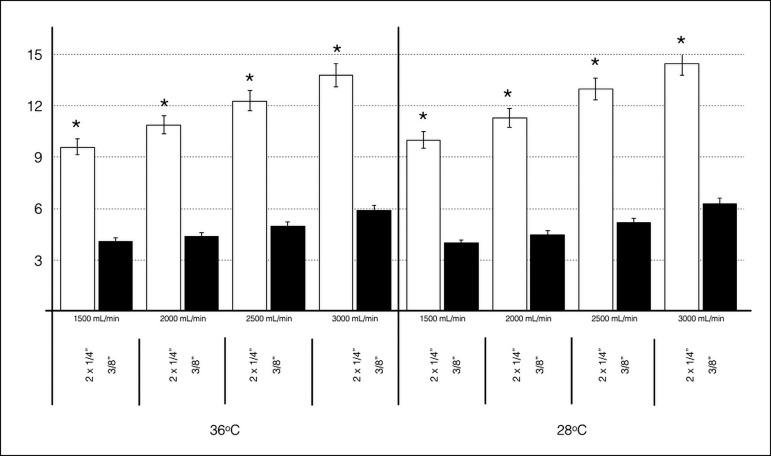
Venous line resistance according to the arterial flow rate observed in Circuits C and D, in both normothermia and hypothermia (setup I), showing significant difference between them (*P<0.001).

The venous line resistance of both Circuits A and B is shown in Figure 3. Arterial line (P3) pressures were maintained at 50 mmHg by a Hoffman clamp during all trials, pre-reservoir pressures increased (became less desirable) at higher flow rates and hypothermia. The difference between the venous line resistance of both Circuits A and B was statistically significant (*P*<0.001). Venous line resistance of both Circuits C and D is shown in Figure 4. The venous line resistance in Circuit C was significantly higher - less desirable - than in Circuit D at higher flow rates and hypothermia; the difference was also statistically significant (*P*<0.001).

## DISCUSSION

All of the CPB circuit components - oxygenator with or without an integrated ALF, venous and cardiotomy reservoirs, A-V cannulae, and tubing - should be evaluated *in vitro* to determine their hydrodynamic performance before they are used in clinical practice^[^^16^^-^^18^^]^. Brazil has a large number of medical devices manufactured and available only in this region which are approved by the National Health Surveillance Agency. These devices commonly do not have large clinical studies comparing clinical data or doing benchmarking of similar devices^[^^19^^-^^21^^]^. In this context, cultural issues associated with the widespread clinical use of devices without any scientific evidence could be responsible for suboptimal outcomes related to perfusion practice. Brazilian manufacturers and international distributors only offer three types of pre-mounted and pre-connected circuits - neonatal, pediatric and adult. There's not the possibility of customizing these circuits for each heart center. Furthermore, oxygenators are sold with a bypass tubing circuit to nearly all cardiac centers around the country. In this framework, the market dictates clinical practice with the common perception that smaller tubing ID is the most important feature when choosing a circuit for small patients. As a point of reference, the neonatal circuit has a dual 3/16" ID venous limb and a single 3/16" ID arterial limb. The pediatric circuit has a dual 1/4" ID venous limb and a single 1/4" ID arterial limb. Furthermore, the adult circuit commonly has a dual 3/8" ID venous limb - even in cases that this might not be needed - and a single 3/8" ID arterial limb.

The use of smaller ID A-V tubing for neonates and infants undergoing CPB procedures is a common perfusion practice in order to minimize the priming volume. However, it is important to remember that smaller ID tubing affects the hemodynamic profiles of CPB circuits, especially when combined with small-sized A-V cannulae for neonates and infants^[^^4^^]^. Adequate venous return is essential to provide the prescribed arterial flow to the patient during CPB. Gravity drainage allows for the movement of blood through the circuit (cannulae and venous limb of bypass circuit), from a higher area (patient on operating room [OR] table) to a lower area (venous reservoir), as long as the fluid column is not interrupted by air. Gravity drainage is dependent on the relative heights of the patient *versus* the venous reservoir, the length and diameters of the venous limb(s), the maintenance of a continuous fluid column, the patient volume status, and CVR characteristics^[^^7^^]^. Smaller CPB circuits may reduce blood bank transfusions at the beginning of CPB run, but if the drainage is suboptimal due to small ID tubing, an extra volume may need to be added to the reservoir to achieve the prearranged pump flow rate. Volume required to keep the venous reservoir volume above the minimum operating level is "dynamic" and may differ from the initial "static" priming volume. Our study shows that there is an insignificant difference in the prime volume of dual venous limb circuits versus a single venous limb circuit. Therefore, the primary consideration becomes the ability to achieve the calculated flow rate with the selected circuit. The findings of this study indicate that the pressure drop in venous limb related to the tubing ID was the main resistance in the venous side of these simulated pediatric CPB circuits. A high resistance in the venous limb (pre-reservoir pressure) may result in insufficient venous return, limiting the perfusionist's ability of maintaining an adequate and safe minimum operating level in the venous reservoir. A higher venous pressure with siphon drainage - less negative-pressure - may require volume addition during CPB which eliminates the initial advantage of a decreased prime volume. As pointed out in our findings, this is the case for dual 3/16" venous limbs when compared to a single 1/4" venous circuit, as well as when comparing dual 1/4" venous limbs to a single 3/8" venous limb.Our results also showed that hypothermia could increase circuit resistance across CPB circuits most probably by increasing the blood viscosity of the perfusate and vascular resistance, which further elevates circuit pressure. Unfortunately, the latter effect cannot be seen in an *in vitro* study due to the fixed compliance of the tubing. Although there was higher (less desirable) pre-reservoir pressure under hypothermia than normothermia, the arterial flow delivered to the pseudopatient was similar.

We intentionally evaluated the circuits at routine CPB pump flow rates along with lower flow rates because the latter may be used during hypothermic CPB and CPB weaning. To be clear, we do not suggest using low flow rates for routine normothermic CPB procedures. For instance, pump flow rates of 500 mL/min can be used during weaning but not during a normothermic full-flow CPB. However, with the same circuit it is possible and it is not uncommon to use high-flow rates during rewarming.

Our data support that a dual 3/16" venous limb may be acceptable but not necessarily practical for venous drainage at a flow up to 1500 ml/min. Ultimately though, a single lower resistance 1/4" venous limb is preferable when compared to a dual 3/16" venous limb at the same arterial flow rate. Finally, a single 3/8" venous limb circuit may be acceptable with gravity drainage at a flow rate up to 3000 ml/min. It is important to note that this experimental design measured overall flow and that clinicians must consider the flow differential between the upper body and lower body when using bicaval cannulation connected independently to dual venous limbs in the pump circuit. It is our hope that these data support a change towards single limb venous circuits which allow for improved achievable flow rates while, at the same time, does not introduce the variable of a limiting dual venous line, which can negatively impact lower body drainage when one limb is connected to the IVC cannula.

### Limitations

Our results can be affected by the fact that this experiment was performed under *in vitro* conditions that may not represent all clinical CPB scenarios. Cannulae selection, table height relative to CVR level, gravity *versus* VAVD, and CVR design impact achievable venous flow rates. Temperatures and flows utilized during congenital heart surgery also vary significantly. Further, individual caval flow may vary considerably in this patient population based on patient's cardiac anatomy.

## CONCLUSION

There was an insignificant difference in priming volume between dual venous and single venous limb circuits. Smaller dual limb venous circuits exhibited a higher venous resistance that was associated with reduced achievable flow and would likely result in impaired venous return during CPB. In addition, impaired venous return with smaller dual limb venous circuits could impose a volume penalty increasing hemodilution in order to keep a safe minimum operating level in the reservoir, which is contrary to the accepted rationale for using smaller ID tubing. Our data indicate that using a single 1/4" venous limb is better than using a dual 3/16" venous limb at all flow rates up to 1500 ml/min flow rate. Moreover, a single 3/8" venous limb is better than a dual 1/4" venous limb up to 3000 ml/min. Assisted venous drainage would improve all values for all circuits, but without any clear benefit since priming volumes are nearly identical.

Our findings strongly suggest a revision of the perfusion practice in Brazil and justify the use of single venous limb circuits for CPB.

**Table:** 

Authors' roles & responsibilities	
LFC	Substantial contributions to the conception or design of the work; or the acquisition, analysis, or interpretation of data for the work; drafting the work or revising it critically for important intellectual content; final approval of the version to be published
GSM	Drafting the work or revising it critically for important intellectual content; final approval of the version to be published
DPG	Substantial contributions to the conception or design of the work; or the acquisition, analysis, or interpretation of data for the work; final approval of the version to be published
GV	Substantial contributions to the conception or design of the work; or the acquisition, analysis, or interpretation of data for the work; final approval of the version to be published
MM	Substantial contributions to the conception or design of the work; or the acquisition, analysis, or interpretation of data for the work; final approval of the version to be published
IC	Agreement to be accountable for all aspects of the work in ensuring that questions related to the accuracy or integrity of any part of the work are appropriately investigated and resolved; final approval of the version to be published
RAN	Drafting the work or revising it critically for important intellectual content; final approval of the version to be published
MBJ	Agreement to be accountable for all aspects of the work in ensuring that questions related to the accuracy or integrity of any part of the work are appropriately investigated and resolved; final approval of the version to be published
SW	SW Substantial contributions to the conception or design of the work; or the acquisition, analysis, or interpretation of data for the work; drafting the work or revising it critically for important intellectual content; final approval of the version to be published
AÜ	Substantial contributions to the conception or design of the work; or the acquisition, analysis, or interpretation of data for the work; drafting the work or revising it critically for important intellectual content; final approval of the version to be published
JCJ	Agreement to be accountable for all aspects of the work in ensuring that questions related to the accuracy or integrity of any part of the work are appropriately investigated and resolved; final approval of the version to be published
FBJ	Agreement to be accountable for all aspects of the work in ensuring that questions related to the accuracy or integrity of any part of the work are appropriately investigated and resolved; final approval of the version to be published
